# ENABLE 2017, the First EUROPEAN PhD and Post-Doc Symposium. Session 4: From Discovery to Cure: The Future of Therapeutics

**DOI:** 10.3390/medsci6020042

**Published:** 2018-05-28

**Authors:** Gianmarco Di Mauro, Ambra Dondi, Giovanni Giangreco, Alexander Hogrebe, Elja Louer, Elisa Magistrati, Meeli Mullari, Gemma Turon, Wouter Verdurmen, Helena Xicoy Cortada, Sanja Zivanovic

**Affiliations:** 1 Institute for Research in Biomedicine (IRB Barcelona), The Barcelona Institute of Science and Technology, Baldiri Reixac 10, 08028 Barcelona, Spain; gemma.turon@irbbarcelona.org (G.T.); sanja.zivanovic@irbbarcelona.org (S.Z.); 2 European School of Molecular Medicine (SEMM), via Adamello 16, 20139 Milano, Italy; ambra.dondi@ieo.it (A.D.); giovanni.giangreco@ieo.it (G.G.); elisa.magistrati@ifom.eu (E.M.); 3 Novo Nordisk Foundation Center for Protein Research, University of Copenhagen, Blegdamsvej 3B, DK-2200 Copenhagen N, Denmark; alexander.hogrebe@cpr.ku.dk (A.H.); meeli.mullari@cpr.ku.dk (M.M.); 4 Radboud Institute for Molecular Life Sciences (RIMLS), Radboud University Medical Center, Geert Grooteplein 28, 6525 GA Nijmegen, The Netherlands; Elja.Louer@radboudumc.nl (E.L.); Wouter.Verdurmen@radboudumc.nl (W.V.); Helena.Xicoy@radboudumc.nl (H.X.C.)

**Keywords:** biomedicine, symposium, therapy, computational biology, cancer, HIV, Alzheimer’s disease, diabetes, nanomaterials

## Abstract

The EUROPEAN ACADEMY FOR BIOMEDICAL SCIENCE (ENABLE) is an initiative funded by the European Union Horizon 2020 program involving four renowned European Research Institutes (Institute for Research in Biomedicine—IRB Barcelona, Spain; Radboud Institute for Molecular Life Sciences—RIMLS, the Netherlands; Novo Nordisk Foundation Center for Protein Research—NNF CPR, Denmark; European School of Molecular Medicine—SEMM, Italy) and an innovative science communication agency (Scienseed). With the aim of promoting biomedical science of excellence in Europe, ENABLE organizes an annual three-day international event. This gathering includes a top-level scientific symposium bringing together leading scientists, PhD students, and post-doctoral fellows; career development activities supporting the progression of young researchers and fostering discussion about opportunities beyond the bench; and outreach activities stimulating the interaction between science and society. The first European PhD and Postdoc Symposium, entitled “Breaking Down Complexity: Innovative Models and Techniques in Biomedicine”, was hosted by the vibrant city of Barcelona. The scientific program of the conference was focused on the most recent advances and applications of modern techniques and models in biomedical research and covered a wide range of topics, from synthetic biology to translational medicine. Overall, the event was a great success, with more than 200 attendees from all over Europe actively participating in the symposium by presenting their research and exchanging ideas with their peers and world-renowned scientists.

## 1. Introduction

**Figure 1:**
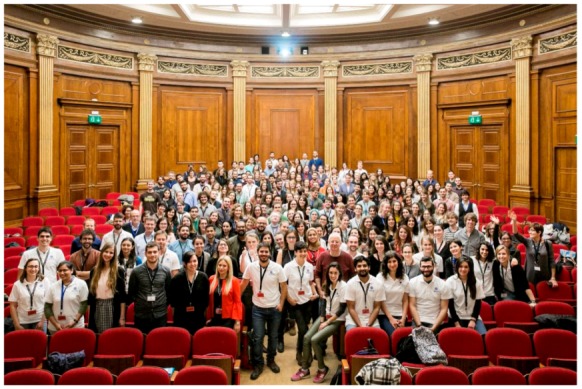
Attendees at ENABLE 2017.

Funded by Horizon 2020, the ENABLE (European Academy for Biomedical Science) project celebrates European research and brings together PhDs and postdocs from all over Europe via activities organized by volunteers and coordinators from the four host institutes—IRB Barcelona, SEMM in Milan, RIMLS in Nijmegen, and CPR in Copenhagen. In November 2017, the first ENABLE conference took place in Barcelona, Spain. The organization of this conference started almost two years earlier and involved a group of 35 volunteer PhD students and postdocs from the four aforementioned research institutes and the support of institute coordinators and the innovative science communication agency, Scienseed. Like many young scientists nowadays, we felt isolated in our own research areas and wanted to build networks beyond our own fields. This is why we launched ENABLE and what the first ENABLE conference also achieved: it involved young scientists opening the academic world from within and promoted crosstalk between disciplines, collaboration with industry, and communication with society at large. The conference in Barcelona was a huge success, with the participation of 272 young researchers from more than 25 countries within the EU and beyond (Figure 1). Companies were also thrilled by our approach, as reflected by our 10 sponsors, who provided more than 60 travel grants, and the more than 20 organizations that were present at our Career Day.

The scientific part of the ENABLE conference was a symposium entitled “Breaking Down Complexity: Innovative Models and Techniques in Biomedicine”. It comprised four sessions, spanning from molecular research to clinical research and potential new therapies. In each session, two distinguished keynote speakers presented their research and gave an overview of their work and the directions their fields are taking. Each keynote lecture was followed by two outstanding presentations by postdocs or PhDs chosen from among the 272 participants. In addition, 100 posters from a range of biomedical fields were presented by the participants, thus facilitating additional discussion between young researchers with diverse backgrounds. Last but not least, the conference also drew society into the discussion by organizing public debates with experts in the field, inviting school children to take part in scientific problem-solving activities at IRB Barcelona, and giving 24 participating young scientists the opportunity to present their work to the public.

## 2. Session 4: From Discovery to Cure: The Future of Therapeutics 

Entitled “From Discovery to Cure: The Future of Therapeutics”, the fourth session of the 2017 ENABLE conference aimed to show cutting-edge techniques that can be applied for the treatment of complex diseases.

The session kicked off with the talk of the keynote speaker Prof. **Eytan Ruppin** (Principal Investigator, Institute for Advanced Computer Studies, University of Maryland, College Park, MD, USA), who described his work on the genome-wide identification of genes that mediate cancer resistance to targeted and immune therapy. After a first phase of response, most types of cancer can eventually acquire resistance to targeted therapies. This resistance is caused by the gain of genomic adaptive alterations that are able to counteract the inactivation of the targeted genes. To circumvent this problem, Prof. Ruppin proposed a computational approach based on the identification of synthetic lethal (SL) genetic interactions, i.e., when the concomitant alteration of two nonessential genes is lethal for the cell. By combining the identification of genes that are inactive exclusively in cancer cells and the inactivation of their SL partners, it would be possible to selectively target malignant cells without damaging non-cancer cells. A similar approach is based on synthetic dosage lethality (SDL), i.e., when a gene becomes essential because of the hyperactivation of another one. Prof. Ruppin’s team developed the DAta-mIning SYnthetic-lethality-identification pipeline (DAISY), which allows the identification of cancer SL and SDL interaction networks on a genome-wide scale. The pipeline analyzes and integrates cancer data from a range of sources, such as somatic copy-number alteration, gene expression, somatic mutations, and gene essentiality data. The resulting SL and SDL interaction networks successfully predicted known SL interactions and novel ones, which have been validated using either siRNAs or drugs. Moreover, the networks predicted the cancer responses to various perturbations, as well as patient survival. In conclusion, the approach used by Prof. Ruppin represents a promising tool for a more effective design of personalized targeted therapies.

**Maria Teresa Zanobio** (PhD student, Department of Molecular Medicine and Medical Biotechnology, University of Naples, Naples, Italy) gave a short talk on the role of the miRNA miR27a as a tumor suppressor in the RS4;11 leukemia cell line. The MLL-AF4 oncogenic chimera, resulting from the t(4;11) chromosomal translocation, is a transcription factor able to drive the neoplastic transformation of hematopoietic progenitors. She found that the expression of miR27a decreased both the MLL-AF4 chimera protein levels and the expression of major MLL-AF4 target genes. Moreover, the expression of miR27a decreased proliferation and increased apoptosis in RS4;11 leukemia cell line. Her work suggests that miR27a represents a potential treatment for t(4;11) acute leukemia.

**Anna Grebinyk** (PhD student, Wildau Technical University of Applied Sciences, Wildau, Germany) gave a short talk on LEDs (Light-Emitting Diodes) as an excitation source for fullerene C_60_ photodynamic cancer therapy. Fullerene is a carbon nanostructure with prooxidant potential; indeed, after photoactivation, it induces reactive oxygen species (ROS) production and, eventually, apoptosis. For the fisrt time the predominant mitochondrial accumulation of pristine fullerene C_60_ in a human leukemic cell line was showed both qualitatively and quantitatively. She analyzed the efficiency of fullerene photodynamics in leukemic cells after exposure to light of different wavelengths provided by high power single chip LEDs. She showed that fullerene was highly phototoxic and induced apoptosis when excited by violet LEDs. The results presented suggest that fullerene C_60_ is a photosensitizer and, importantly, that it can be used in combination with LEDs as light source for cancer treatment.

The second keynote speaker of the session was Dr. **Christian Brander** (Principal Investigator, Institut de Recerca de la Sida, Barcelona, Spain), who described his work on predictors of HIV control and their use for HIV vaccine design. The most effective way to control and fight the HIV virus is to create a specific vaccine. However, several factors limit the development of an effective vaccine, including the different variants of the virus and the genetic variance of the host. Indeed, the host genetic background determines responses to the infection, ranging from individuals that can control it even in the absence of antiretroviral treatment, to others that progress to full-blown AIDS within one or a few years after infection. Understanding the mechanisms that mediate in vivo viral control is important for the development of effective vaccines. To this end, Dr. Brander’s group developed and applied more sensitive analytic tools for the definition of biomarkers of controlled HIV infection, focusing on signaling proteins and cell-to-cell communication factors detectable from peripheral blood samples. By comparing the profiles of HIV-infected individuals with different levels of viremia, they identified novel markers of HIV control and disease progression, such as specific cytokine patterns and individual soluble markers. Among these, interleukin-27 (IL-27) emerges as a predictor of viral load and blood viral reservoir size. The results presented by Dr. Brander open up the possibility of understanding the molecular mechanisms of relative HIV control and HIV resistance, which are crucial for the development of an effective HIV vaccine.

**Rita Garcia Ribeiro** (PhD student, Biomedical MRI/MoSAIC, Department of Imaging and Pathology, Biomedical Sciences Group, KU Leuven, Leuven, Belgium) presented a short talk on the use of magnetic resonance imaging (MRI) for real-time in vivo monitoring of transplanted pancreatic islets in a diabetic rat model. Pancreatic islet transplantation emerges as a potential treatment for type I diabetes mellitus, but has shown low efficiency so far. To monitor functional islet grafts in real-time, she proposed an innovative strategy based on MRI of pancreatic islets labeled with ultra-small paramagnetic iron oxide (USPIO). The main advantages of this approach are the non-invasiveness of the tracking, the high efficiency of cell labeling without toxic effects, and the stability of MRI contrast over time. Transplantation of USPIO-labeled islets in healthy and diabetic rats followed by MRI demonstrated the efficiency and stability of the labeling, as well as the low toxicity for the host. Her work supports MRI of USPIO-labeled islets as a valuable tool for clinical assessment of the efficiency of islet grafting.

**Sofía Fernández-de-Retana** (PhD student, Neurovascular Research Laboratory, Vall d’Hebron Research Institute, Universitat Autònoma de Barcelona, Spain) presented a short talk on the characterization of ApoJ-reconstituted high-density lipoprotein (rHDL) nanodiscs for the potential treatment of cerebral β-amyloidosis. The accumulation of β-amyloid protein in the brain is a pathological feature of Alzheimer’s disease. To avoid cerebral β-amyloidosis, high-density lipoprotein (HDL)-based therapies have been proposed. In particular, the proposed strategies consist of using reconstituted rHDL-rApoJ nanoparticles by assembling phospholipids with recombinant human Apolipoprotein J (ApoJ). ApoJ is a natural chaperone that interacts with β-amyloid protein and prevents its aggregation and toxicity. She showed that rHDL-rApoJ particles prevented β-amyloid protein fibrillization and mediated a higher cholesterol efflux in cultured cells. Moreover, rHDL-rApoJ particles accumulated in the cranial region, especially in aged transgenic mice presenting a high cerebral β-amyloid protein load. Therefore, on the basis of these results, rHDL-rApoJ was put forward as a potential therapeutic option for β-amyloid-related pathologies.

## 3. Conclusions and Future Perspectives of ENABLE

The first ENABLE conference was a success: 35 PhD and postdoc volunteers from four European research institutes, with support from the institute coordinators and Scienseed, organized an event in which 272 young researchers from over 25 countries presented and shared their science and experiences in scientific talks, poster sessions, master classes, general public talks, and evening activities. More than 60 attendees were given the opportunity to participate through the award of a travel grant funded by one of our 10 sponsors. The symposium, entitled “Breaking Down Complexity: Innovative Models and Techniques in Biomedicine”, was created to cover a broad range of topics in biomedical research, to encourage participation and the exchange of ideas, and to promote future collaborations among young scientists.

In order to include multiple research areas, there was a scientific program with four sessions. The first, entitled “Building the Foundations of Biology: Synthetic and Cellular Research”, featured Prof. Martin Hanczyc (University of Trento, Italy) and Prof. Elaine Fuchs (The Rockefeller University, New York, NY, USA) as keynote speakers and included short talks on DAPK regulation, nanoscale redistribution of NMDA receptors in autoimmune encephalitis, targets associated with metabolic reprogramming in hematological malignancies, and the mechanisms regulating aortic arch development. The second session, “The OMICS Revolution: Understanding the Layers of Life”, featured Prof. Johan Auwerx (Ecole Polytechnique, Lausanne, Switzerland) and Prof. Ruedi Aebersold (ETH Zurich, Switzerland) as keynote speakers and included short talks on proteomics to study the role of PRC2 in embryonic stem cells, single-cell sequencing to reconstruct the cell lineages of whole adult animals, the effects of endocrine-disrupting chemicals on development, and on quantitative proteomics to study fibrotic networks. The third session, “In Vitro to in Vivo: Modeling Life in 3D”, featured Prof. Kristina Havas Cavalletti (IFOM, Milan, Italy) and Dr. Kim Jensen (BRIC, Copenhagen, Denmark) as keynote speakers and included short talks on gut vascular barrier disruption and type 2 diabetes, the link between metabolic dysfunction and immune complications in lysinuric protein intolerance, the role of obesity in the development of acute promyelocytic leukemia, and the TGF-β pathway in colorectal cancer metastasis. The fourth and final session, “From Discovery to Cure: The Future of Therapeutics”, featured Prof. Eytan Ruppin (Univeristy of Maryland, CBCB, College Park, MD, USA) and Prof. Christian Brander (IrsiCaixa, Barcelona, Spain) as keynote speakers and included short talks on the role of miR27a as a tumor suppressor, photodynamic cancer therapy, real-time in vivo monitoring of transplanted islets, and a high-density lipoprotein nanodisc for the potential treatment of cerebral β-amyloidosis.

The success of the event was confirmed by the satisfaction scores (out of 5) given by the participants. In this regard, they gave the 2017 ENABLE symposium 4.4, the general topic of the symposium 4.1, and the keynote talks 4.4. The favorite part of the symposium was “Tapas with the Speakers” (score of 4.5), an activity that allowed the participants to interact with the keynote speakers in an informal setting while enjoying some typical Spanish food. The satisfaction of the attendees was also reflected by comments made on the evaluation form, such as “The option of travel grants is amazing and the general idea of the symposium is great. Great speakers, amazing food, nice event. Congratulations”, “I think that the ENABLE project is an amazing idea. It is a little bit different than other conferences because of the career day. The fact that it was organized by PhD students is really interesting!” and “On the all, the symposia was super nice and it was extremely great to really discuss science on a reality level. No one wanted to show off or pretended to be the best scientist in the world and this was awesome”.

To conclude, the symposium brought together renowned scientists with young scientists and can be considered a huge success. The enthusiasm of the participants and the positive feedback received after the event underscore this notion and indicate that the ENABLE conference series has got off to an excellent start, with all eyes now focused on the 2018 event in Copenhagen.

The second symposium of the ENABLE series will be hosted by the Novo Nordisk Foundation Center for Protein Research (CPR, University of Copenhagen, Denmark), one of the partner institutions of the ENABLE consortium. It will take place 6–9 November 2018 at the Maersk Tower in Copenhagen. 

Entitled “The Promise of Future Medicine: From Research to Therapy”, the symposium will explore state-of-the-art biomedical research from basic science to clinical practice and patient outcome. By bringing together 300 PhD students and postdocs, as well as nine eminent keynote speakers from diverse research fields, the next ENABLE symposium seeks to foster a multidisciplinary environment and crosstalk between biomedical disciplines. The following speakers have already confirmed their participation: Helen Lee (Cambridge University, Cambridge, UK); Giuseppe Testa (European Institute of Oncology, Milan, Italy); Nazneen Rahman (Institute of Cancer Research, London, UK); Klaus Pantel (Institute of Tumour Biology, University Medical Centre Hamburg-Eppendorf, Hamburg, Germany); Michel Morange (IHPST, Paris, France); Andrea Bertotti (IRCCS Candiolo, Italy); and Matthew Wood (University of Oxford, Oxford, UK). 

Apart from the scientific symposium, a Career Day is foreseen, to allow participants to broaden their career perspectives. This activity will involve chats with professionals, high-quality workshops, and an Opportunity Fair, which will allow participants to come into direct contact with companies belonging to a variety of sectors. To support the participation of young researchers from all over Europe, our sponsors will provide about 40 travel grants to cover the registration fee and travel and accommodation expenses. Up-to-date information on the event can be found on our website (https://enablenetwork.eu/).

We look forward to the 2018 symposium and are confident that ENABLE will foster the establishment of a network that promotes efficient and synergistic scientific exchange among researchers throughout Europe.

